# PS-38 - OUTBREAK OF HEPATITIS A IN LANARKSHIRE WITH THREE DISTINCT CLUSTERS: INVESTIGATION AND CONTROL MEASURES

**DOI:** 10.1093/pubmed/fdag046.074

**Published:** 2026-07-09

**Authors:** Ms Alison Smith-Palmer, Dr Varon Lopez Carlos Varon Lopez, Ms Susan Frew, Dr Samantha Shepherd, Prof Rory Gunson, Mr Stephen Dufffy, Ms Carolynne Coole-Miller, Dr Sharon Zahra, Dr Nicole Priddee, Dr Lisa Javis, Ms Jacqueline Trollan, Ms Joanne Miller, Ms Susan Brennan, Ms Roxanna Hendry, Ms Irene Morrison Morrison, Ms Elizabeth Grey, Ms Bee Hunter, Dr Henry Prempeh, Ms Jill Bell, Dr Sema Nickbakhsh, Dr Lynda Browning

**Affiliations:** NHS Lanarkshire; NHS Lanarkshire; NHS Lanarkshire; West of Scotland Specialist Virology Centre; West of Scotland Specialist Virology Centre; North Lanarkshire Environmental Health Department; North Lanarkshire Education Department; Scottish National Blood Transfusion Service; Scottish National Blood Transfusion Service; Scottish National Blood Transfusion Service; NHS Lanarkshire; NHS Lanarkshire; NHS Lanarkshire; NHS Lanarkshire; North Lanarkshire Environmental Health Department; North Lanarkshire Environmental Health Department; North Lanarkshire Education Department; NHS Lanarkshire; NHS Lanarkshire; Public Health Scotland; Public Health Scotland

## Abstract

**Background:**

Between February and September 2024 NHS Lanarkshire managed an outbreak of Hepatitis A (HAV) which occurred in three distinct clusters focused in one town. Outbreaks HAV are relatively infrequent events, but when they do occur urgent action is required to identify close contacts, chains of transmission and provide vaccination and where required immunoglobulin to close contacts and exclusion when required for some contacts in high risk occupations.

**Objectives:**

Prevent onward transmission of HAV

**Methods:**

Outbreak Management included vaccination at two nurseries, a primary school and ASN school.

**Results:**

A total of 20 confirmed cases across the three clusters, 11 of which required hospitalisation. Epidemiological links were identified between all three clusters, and chains of transmission identified within each cluster. Cases clustered genetically based on molecular typing. Molecular typing did not identify cases elsewhere in the UK supporting hypothesis of local chains of person to person transmission. As the outbreak evolved the criteria for close contacts and those eligible for vaccination was extended to include social contacts and the household members of staff/pupils offered vaccination in one of the nurseries and the ASN school. Over 600 individuals were vaccinated to control the outbreak.

**Conclusions:**

Challenges included the tight timescales for establishing clinics, ensuring a high uptake rate and extensive contact tracing. Testing was expanded to some asymptomatic individuals which supported case ascertainment especially among children who were often identified as likely sources of infection for others within the clusters.

Consideration was also given to the potential of onwards transmission through the cases having been donors of substances of human origin (SoHO) such as donation of blood or bone. This prompted liaison with the Scottish National Blood Transfusion Service to make sure that the supply chain of SoHO was not compromised during the outbreak

